# Estimating the economic impact of comorbidities in patients with MASH and defining high-cost burden in patients with noncirrhotic MASH

**DOI:** 10.1097/HC9.0000000000000488

**Published:** 2024-07-22

**Authors:** Zobair M. Younossi, Kamal Kant Mangla, Abhishek Shankar Chandramouli, Jeffrey V. Lazarus

**Affiliations:** 1 The Global NASH Council, Washington, District of Columbia, USA; 2 Beatty Liver and Obesity Research Program, Inova Health System, Falls Church, Virginia, USA; 3 Novo Nordisk Service Center India Pvt Ltd, Bangalore, Karnataka, India; 4 City University of New York Graduate School of Public Health and Health Policy (CUNY SPH), New York, New York, USA; 5 Barcelona Institute for Global Health (ISGlobal), Hospital Clínic, University of Barcelona, Barcelona, Spain

## Abstract

**Background:**

Metabolic dysfunction–associated steatohepatitis (MASH) is associated with high health care costs. This US study investigated the economic burden of MASH, particularly in patients without cirrhosis, and the impact of comorbidities on health care costs.

**Methods:**

This retrospective, observational study used data from patients diagnosed with MASH aged ≥18 years from October 2015 to March 2022 (IQVIA Ambulatory electronic medical record-US). Patients were stratified by the absence or presence of cirrhosis. Primary outcomes included baseline characteristics and annualized total health care cost after MASH diagnosis during follow-up. In addition, this study defined high costs for the MASH population and identified patient characteristics associated with increased health care costs among those without cirrhosis.

**Results:**

Overall, 16,919 patients (14,885 without cirrhosis and 2034 with cirrhosis) were included in the analysis. The prevalence of comorbidities was high in both groups; annual total health care costs were higher in patients with cirrhosis. Patients with a high-cost burden (threshold defined using the United States national estimated annual health care expenditure of $13,555) had a higher prevalence of comorbidities and were prescribed more cardiovascular medications. MASH diagnosis was associated with an increase in cost, largely driven by inpatient costs. In patients without cirrhosis, an increase in cost following MASH diagnosis was associated with the presence and burden of comorbidities and cardiovascular medication utilization.

**Conclusions:**

Comorbidities, such as cardiovascular disease and type 2 diabetes, are associated with a higher cost burden and may be aggravated by MASH. Prioritization and active management may benefit patients without cirrhosis with these comorbidities. Clinical care should focus on preventing progression to cirrhosis and managing high-burden comorbidities.

## INTRODUCTION

NAFLD (now known as metabolic dysfunction–associated steatotic liver disease [MASLD]^1^) encompasses a spectrum from steatotic liver, without or with minimal inflammation,^2^ to its progressed form NASH (now known as metabolic dysfunction–associated steatohepatitis [MASH]^1^), which is present in ~5% of the United States general population and is significantly underdiagnosed.^3^,^4^,^5^ Progression to MASH involves steatosis in combination with inflammation and evidence of hepatocyte injury (such as ballooning), with or without fibrosis, which may eventually result in irreversible cirrhosis.^2^ MASH may be asymptomatic or present with symptoms that are not liver-specific until complications develop.^6^,^7^,^8^ Currently, the gold standard diagnosis relies on liver biopsy,^8^ the invasive nature of which can result in delayed diagnosis, injury, and increased costs.^8^,^9^,^10^

MASH is associated with liver-related complications, such as end-stage liver disease and hepatocellular carcinoma, as well as comorbidities, such as CVD, obesity, and type 2 diabetes.^2^,^9^,^11^ Recently, there has been an increased focus on the costs and health care burden associated with MASLD/MASH.^12^,^13^,^14^,^15^,^16^,^17^ Research in a large population with MASLD showed an increase in costs following diagnosis compared with a control group of similar age and similar comorbidities, with diagnosis also leading to an increase in health care utilization, especially in the first year.^18^ In the United States, lifetime costs for all patients with MASH were estimated at $222.6 billion in 2017 and $95.4 billion for patients with advanced MASH.^15^ In Europe, the total economic costs of MASH have been estimated to be between €8.5 and 19.5 billion.^13^

Despite growing evidence regarding the high health care costs and utilization associated with MASH, there remains an unmet need to better understand how MASH severity (including the absence or presence of cirrhosis) and the presence of associated comorbidities affect health economics. It is estimated that there are >18 million people in the United States living with type 2 diabetes and MASLD, of whom >6 million have MASH; 20-year costs for MASLD in these patients were calculated to exceed $55 billion.^16^ Existing health economic models suggest that the greatest absolute costs are incurred by patients with MASLD, who represent the greatest number of patients overall, rather than those with more advanced MASH.^14^,^19^ Thus, understanding the burden in those without cirrhosis could improve patient management and treatment approaches. Furthermore, there is evidence that the presence of MASLD and MASH influences the risk and severity of other conditions, such as type 2 diabetes and CVD, and vice versa.^20^,^21^,^22^ It is therefore important to understand how comorbidities in those without cirrhosis may affect economic burden.

Therefore, the aim of this study was to understand and quantify the economic burden associated with MASH in those without and with cirrhosis, along with analyzing how comorbidities impact the economic burden. In addition, we aimed to define and characterize specific patient profiles in those without cirrhosis that were associated with a high economic burden.

## METHODS

### Study design and patient population

**FIGURE 1:**
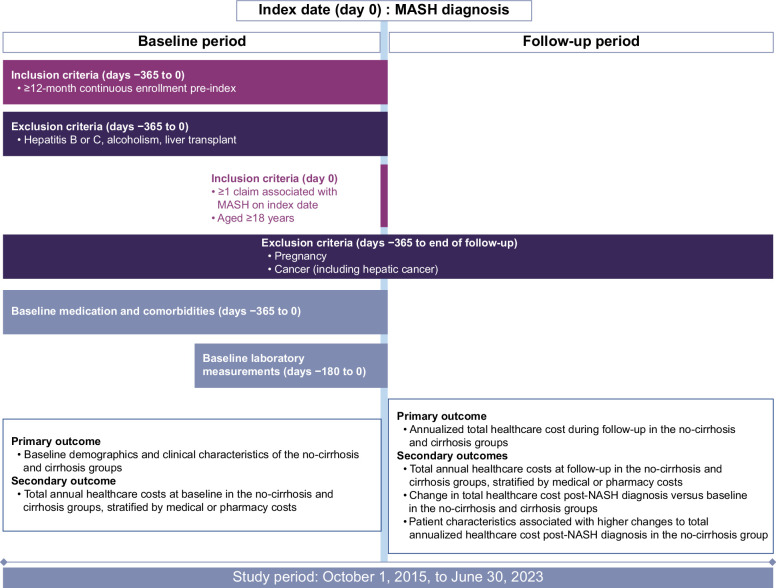
Study design. For laboratory measurements, the closest value in the 6 months before the index date was used. The time window to select biomarkers was +30 days from the index date in follow-up. The figure was developed using guidance on the graphical representation of longitudinal studies.^24^ Abbreviation: MASH, metabolic dysfunction–associated steatohepatitis; NASH, nonalcoholic steatohepatitis.

In this retrospective, observational study, anonymized data from a large, commercially available US health care data set (IQVIA Ambulatory electronic medical record-US with linked PharMetrics Plus claims), which collates data from physician practices for ~71 million patients,^23^ were analyzed. Patients aged ≥18 years who had at least 1 claim associated with MASH diagnosis (identified using the ICD-10 code, K75.81) between October 1, 2015, and June 30, 2023, were included (Figure 1). The index date was defined as the first identified date of MASH diagnosis. The presence of cirrhosis was defined as having an ICD-9 or ICD-10-CM diagnosis related to cirrhosis (ICD-9: 571.5, 571.6, 571.9; ICD-10-CM: K74.0–74.6). Data in the 12 months before MASH diagnosis were used as baseline data for the analysis, while data from the date of diagnosis to the end of the study period (March 31, 2022) were analyzed as follow-up data.

For laboratory measurements, such as glycated hemoglobin and body mass index, the closest value in the 6 months before the index date was used. The Fibrosis-4 index (FIB-4) score was calculated as age (y) × aspartate aminotransferase (U/L)/(platelets [10^9^/L] × alanine aminotransferase [U/L]^1/2^) and categorized as low (<1.30), intermediate (1.30–2.67), or high (>2.67) risk of advanced fibrosis using cutoffs shown to be associated with advanced fibrosis and in alignment with clinical guidelines.^25^,^26^,^27^,^28^ Baseline comorbidities were defined as conditions that had been diagnosed in the 12 months before the index date.

Patients were required to have continuous enrollment in the 12-month baseline period. The follow-up period for each patient was from the index until the last date of continuous enrollment. Patients were included irrespective of the length of follow-up available. Continuous enrollment was used to understand the available follow-up duration and was defined using the claims part of the database based on the patients’ insurance enrollment. Reasons for discontinuing the enrollment plan were not available in the databases and could have been due to multiple reasons, including loss to follow-up in the database and/or mortality. Patients were censored at the time of loss of follow-up.

Patients with a diagnosis of hepatitis B or C, diagnostic evidence indicating alcohol use, dependency, or related disorders, or liver transplant, at any time in the baseline period, and patients with a diagnosis of any cancer, including hepatic cancer, or evidence of pregnancy during the study period were excluded. Patients were stratified by the presence of cirrhosis in the 12-month baseline period or at the time of index into cirrhosis and no-cirrhosis groups.

All research was conducted in accordance with the Declarations of Helsinki and Istanbul. This retrospective study used deidentified health claims data and was thus exempt from institutional review board review and the requirement for informed consent. The study complied with all applicable laws regarding patient privacy, using compliant deidentified data sources, and the research is reported in line with the Strengthening the Reporting of Observational Studies in Epidemiology (STROBE) guidelines for cohort studies.

### Outcomes

#### Primary outcomes

The primary outcomes were the baseline demographics and clinical characteristics of the cirrhosis and no-cirrhosis groups from the 12-month period prior to the index. These data included the prevalence of comorbidities identified based on ICD-9 and ICD-10-CM coding and claims associated with concomitant medications. A list of ICD codes used to define CVD-related comorbidities can be found in Supplemental Table S1, http://links.lww.com/HC9/A977. It was assumed that the medications from claims were used and taken as indicated, specifically those related to glucose-lowering in patients with type 2 diabetes and those related to cardiovascular (CV) treatment in patients with CVD, which was assessed at class level using pharmacy claims in the baseline period.

Another primary outcome was the annualized total health care cost incurred during follow-up for both the cirrhosis and no-cirrhosis groups, including the proportion of patients in different thresholds of total health care cost burden: ≥$5000 per annum, ≥$15,000 per annum, and ≥$50,000 per annum. Furthermore, patients in both groups were stratified as having either a high-cost burden or a non-high–cost burden. A high-cost burden was defined as an annual total health care cost higher than the average medical costs in the United States, that is, $13,555 per capita per annum, based on data from the National Health Expenditure Accounts in the United States (Centers for Medicare & Medicaid Services 2022, adjusted for inflation to 2023 values). Patients in the no-cirrhosis group were further characterized to identify baseline comorbidities and concomitant medications prevalent in those with a high-cost burden.

#### Secondary outcomes

Secondary outcomes for the cirrhosis and no-cirrhosis groups were the total annual health care costs at baseline and in follow-up stratified by medical (inpatient or outpatient) or pharmacy costs, along with the change in total health care cost following MASH diagnosis from baseline. Patient characteristics (such as absence vs. presence of comorbidities and their severity) associated with higher changes in total annualized health care cost post-MASH diagnosis was a secondary outcome in the no-cirrhosis group. Severity was assessed using the Quan-Charlson Index (QCI), a measure of the risk of mortality associated with comorbidities, the adapted Diabetes Complications Severity Index (aDCSI), and the use of glucose-lowering and CV-related medications (patients with higher numbers of baseline glucose-lowering and CV-related drug classes were assumed to have higher severities of type 2 diabetes and CVD).

#### Health care expenditure by disease cause

Total health care cost was analyzed by disease cause as liver-related, CV-related, liver- and CV-related, and other (non-CV and non-liver–related). The following criteria were used to define the disease cause related to the claim(s): If any of the diagnoses (not just primary) or procedures corresponded to liver and did not have any CV-related codes, then the claim was classified as liver-related cost; CV and did not have any liver-related codes, then the claim was classified as a CV-related cost; both liver- and CV-related codes, then the claim was classified as a liver- and CV-related cost; any claim not falling into the above categories then the claim was classified as an “other” cost.

### Statistical analysis

Descriptive statistics were used to describe baseline characteristics of patients (mean with SD and median with IQR [Q1, Q3] for continuous variables; number and proportion of patients for categorical variables). The number of patients with data was reported for each variable. Missing data were not imputed. Fisher exact test of independence and the *t* test were performed for categorical and continuous measures, respectively, to check for statistically significant differences in baseline demographics and clinical characteristics between the cirrhosis and no-cirrhosis groups. Statistical tests for the endpoints were performed as 2-sided tests, with a significance level of 0.05. Differences in characteristics in patients with a high-cost burden versus non-high–cost burden were assessed using Fisher exact test for categorical variables and the Wilcoxon signed-rank test for continuous variables.

Health care cost in follow-up was estimated based on claims data and included records from the index date until the end of continuous enrollment. The estimated cost was then annualized as patients had variable lengths of follow-up data available. Total annualized health care follow-up costs and change in total annualized health care costs after MASH diagnosis were calculated in US dollars ($) and reported as mean (SD) and median (Q1, Q3). Statistical difference in total health care cost in follow-up was analyzed using a generalized linear model with a gamma distribution and log link function, with cost as the dependent variable and cirrhosis versus no-cirrhosis group as the independent covariate. The available length of follow-up was included as offset. Statistical difference was assessed after adjusting for key baseline characteristics that could impact the outcomes, including age at index date, sex, US region, QCI, aDCSI, insurance plan type (commercial or Medicare), previous glucose-lowering treatment, previous CV-related medication, comorbid conditions, and baseline costs.

Change in total health care cost from baseline to follow-up was defined as: (annualized cost of patients post-MASH diagnosis) − (annualized cost of patients pre-MASH diagnosis). The effect of baseline characteristics on the change in costs was assessed using a multivariate generalized linear model with a gamma distribution and log link function. The available length of follow-up was included as offset. Covariates with a *p* value<0.05 were considered as characteristics impacting change in total health care cost. The total follow-up health care costs for the no-cirrhosis group were additionally analyzed by type and total number of comorbidities: the least-square means outputs and β coefficients were used to define the effect of covariates of cost in terms of percentage.

The list of covariates included in the multivariate model to understand the effect on change in cost was based on significance assessed using the generalized linear model univariate analysis. Covariates included QCI, aDCSI, insurance plan type (commercial or Medicare), US region, previous glucose-lowering treatment, previous CV-related medication, and comorbid conditions. The cost was transformed to the fifth root for a better model fit.

## RESULTS

### Population

A total of 16,919 patients were included in the analysis, 14,885 (88%) and 2304 (12%) in the no-cirrhosis and cirrhosis groups, respectively. Baseline demographics and clinical characteristics are presented in Table 1. In summary, the mean (SD) age was 52.1 (11.7) years, and 53% of patients were female. Median (Q1, Q3) follow-up was 19 (0, 92) and 15 (0, 92) months for the no-cirrhosis and cirrhosis groups, respectively. FIB-4 scores were available for 745 patients, and the mean score was 1.69 (1.72); the mean FIB-4 score was significantly higher in the cirrhosis group compared with the no-cirrhosis group (3.60 vs. 1.38, respectively; *p* < 0.0001). Of 745 patients with an FIB-4 score, the proportion of patients in the high (>2.67), intermediate (1.30–2.67), and low (<1.30) baseline FIB-4 risk groups was 14.5% (108 patients), 29.3% (218 patients), and 56.2% (419 patients), respectively. There was a greater proportion of patients in the cirrhosis groups versus the no-cirrhosis group with a high risk of advanced fibrosis (FIB-4 >2.67): 3% versus 0%, respectively. The NAFLD fibrosis score was significantly higher in the cirrhosis group compared with the no-cirrhosis group (1.20 [94 patients] vs. –1.46 [580 patients], respectively; *p* < 0.0001).

**TABLE 1:** Patient demographics and clinical characteristics

Demographic	All patients (N=16,919)	No cirrhosis (N=14,885)	Cirrhosis (N=2034)
Age, y			
Mean (SD)	52.12 (11.66)	51.25 (11.60)	58.49 (9.95)
Median (Q1, Q3)	54 (45, 61)	53 (44, 60)	60 (53, 64)
Female, %	53	52	58
MASH-relevant biomarkers			
APRI***, n	965	829	136
Mean (SD)	0.62 (0.82)	0.55 (0.70)	1.09 (1.24)
Median (Q1, Q3)	0.40 (0.24, 0.69)	0.37 (0.23, 0.62)	0.72 (0.38, 1.36)
Fibrosis-4 index***, n	745	638	107
High (>2.67), n	108	54	54
Intermediate (1.30–2.67), n	218	183	35
Low (<1.30), n	419	401	18
Mean (SD)	1.69 (1.72)	1.38 (1.17)	3.60 (2.87)
Median (Q1, Q3)	1.16 (0.75, 1.88)	1.05 (0.71, 1.56)	2.71 (1.69, 5.08)
NAFLD fibrosis score***, n	674	580	94
Mean (SD)	–1.09 (1.87)	–1.46 (1.60)	1.20 (1.80)
Median (Q1, Q3)	–1.23 (–2.30, 0.00)	–1.49 (–2.51, –0.47)	1.36 (0.22, 2.45)
Other clinical measures			
QCI***			
Mean (SD)	2.03 (1.71)	1.73 (1.43)	4.23 (1.99)​
Median (Q1, Q3)	2 (1, 3)	2 (0, 2)	4 (3, 5)
aDCSI***			
Mean (SD)	0.86 (1.48)	0.72 (1.30)	1.91 (2.14)​
Median (Q1, Q3)	0 (0, 1)	0 (0, 1)	1 (0, 3)
HbA1c, n	754	641	113
Mean, % (SD)	6.81 (1.73)	6.76 (1.71)	7.29 (1.92)
Median, % (Q1, Q3)	6.3 (5.6, 7.4)	6.2 (5.6, 7.4)	6.8 (6.0, 8.3)
BMI, n	3549	3082	467
Mean, kg/m^2^ (SD)	34.27 (6.62)	34.18 (6.58)	34.88 (6.82)
Median, kg/m^2^ (Q1, Q3)	33.85 (29.68, 39.01)	33.72 (29.60, 38.88)	34.72 (30.27, 40.01)

*** *p* < 0.0001 for no cirrhosis versus cirrhosis.

Abbreviations: aDCSI, adapted Diabetes Complications Severity Index; APRI, aspartate aminotransferase to platelet ratio; HbA1c, glycated hemoglobin; MASH, metabolic dysfunction–associated steatohepatitis; QCI, Quan-Charlson Index.

### Comorbidities and prescribed medications at baseline

**FIGURE 2:**
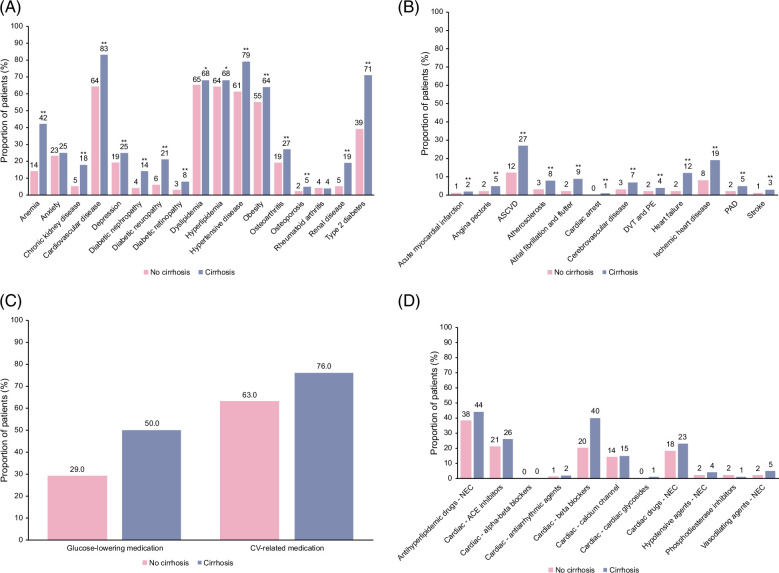
Prevalence of (A) overall baseline comorbidities, (B) cardiovascular-related comorbidities, (C) glucose-lowering and cardiovascular-related medications, and (D) cardiovascular-related medication by class. Glucose-lowering medications include amylin analogs, biguanides, sulfonylureas, sodium-glucose cotransporter-2 inhibitors, dipeptidyl peptidase-4 inhibitors, alpha-glucosidase inhibitors, combination oral therapies, “other” (glucagon and diazoxide), insulin-sensitizing agents, meglitinide analogs, dopamine receptor agonists, glucagon-like peptide-1 receptor agonists, and insulin. Obesity classified as diagnosed or measured BMI ≥30 kg/m^2^, ASCVD is defined as the presence of at least 1 out of the following: atherosclerosis, angina pectoris, PAD, cerebrovascular disease, or ischemic heart disease. *p<0.05 cirrhosis versus no-cirrhosis groups, **p<0.0001 cirrhosis versus no-cirrhosis groups. A list of ICD-9-CM and ICD-10-CM codes is available in the Supplemental Digital Content, http://links.lww.com/HC9/A977. Abbreviations: CV, cardiovascular; NEC, not elsewhere classified.

The most prevalent comorbidities in the no-cirrhosis group were hyperlipidemia/dyslipidemia (65%), hypertension (63%), and obesity (56%). The most prevalent comorbidities in the cirrhosis group were hypertension (79%), type 2 diabetes (71%), and hyperlipidemia/dyslipidemia (68%). The prevalence of all individual comorbidities, except for anxiety and rheumatoid arthritis, was significantly higher in the cirrhosis versus no-cirrhosis group (*p*<0.005) and was numerically higher in the cirrhosis versus no-cirrhosis group for anxiety (Figure 2A). QCI and aDCSI scores were significantly higher in the cirrhosis versus no-cirrhosis group (*p*<0.0001; Table 1). Regarding CV-related comorbidities (Figure 2B), the cirrhosis group had a significantly higher prevalence of acute myocardial infarction, angina pectoris, atherosclerosis, atherosclerotic CVD, atrial fibrillation and flutter, cardiac arrest, cerebrovascular disease, deep vein thrombosis and pulmonary embolism, heart failure, ischemic heart disease, peripheral artery disease, and stroke (all *p*<0.0001).

The proportion of patients prescribed glucose-lowering and CV-related medications at baseline was also high in both groups, with most patients having claims related to CV-related medications (Figures 2C, D). The use of glucose-lowering and CV-related medications was higher in the cirrhosis versus no-cirrhosis group (50% vs. 29% for glucose-lowering medication and 76% vs. 63% for CV-related medication, respectively).

### Annualized total follow-up health care cost burden

The overall annualized mean (SD) follow-up cost for all patients was $46,662 (928,285), and costs were more than twice as high for the cirrhosis versus no-cirrhosis group ($98,574 [432,465] vs. $39,568 [976,477], respectively). Median (Q1, Q3) follow-up costs were $10,701 (3850, 28,849) for all patients, $9471 (3471, 24,296) for the no-cirrhosis group, and $29,352 (10,784, 78,071) for the cirrhosis group. Mean follow-up costs remained higher for the cirrhosis versus no-cirrhosis group after adjustment for age, sex, US region, insurance type, and comorbidities (*p*<0.0001).

**FIGURE 3:**
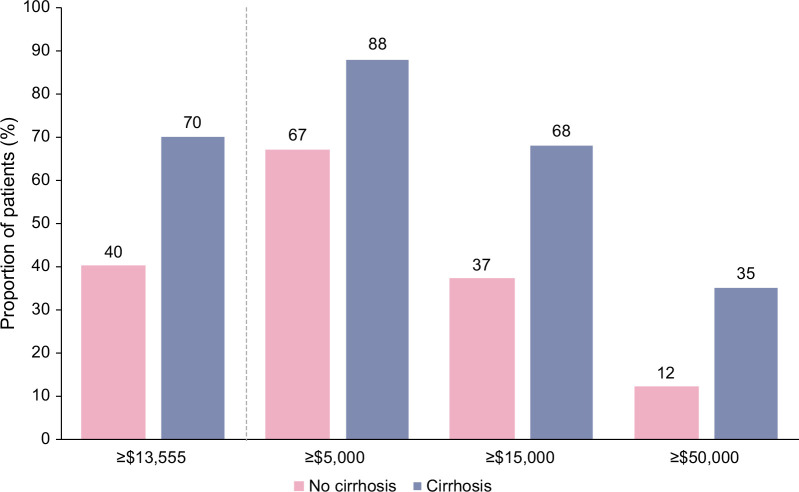
Cost stratification according to different thresholds.

Stratification of annualized mean follow-up costs into categories of ≥$5000, ≥$15,000, and ≥$50,000 is presented in Figure 3. The proportion of patients with cost burdens of ≥$5000, ≥$15,000, and ≥$50,000 in the no-cirrhosis and cirrhosis groups was 67% and 88%, 37% and 68%, and 12% and 35%, respectively. A total of 40% of patients in the no-cirrhosis group and 70% of patients in the cirrhosis group had a high-cost burden (≥$13,555; Figure 3).

### Patient characteristics associated with high-cost burden in the no-cirrhosis group

**FIGURE 4:**
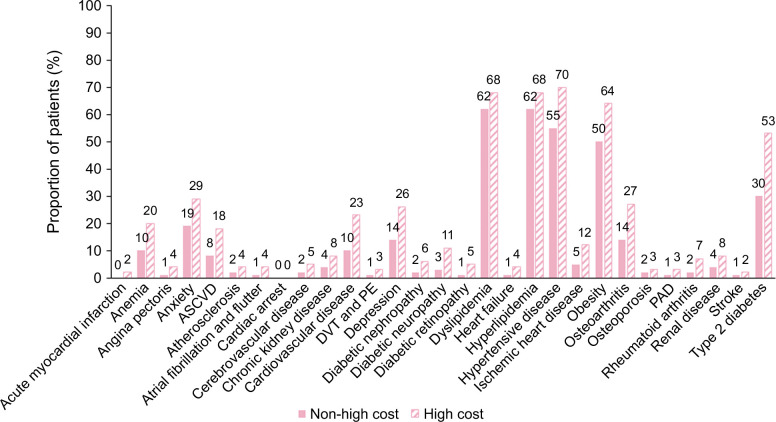
Prevalence of baseline comorbidities for patients in the no-cirrhosis group with high-cost burden (≥$13,555) and non-high–cost burden (<$13,555). High cost was defined as ≥$13,555, according to the United States national estimate on annual health care expenditure. All differences between non-high–cost and high-cost measurements were significant (*p* < 0.0001 unless otherwise stated). Obesity classified as diagnosed or measured BMI ≥30 kg/m^2^. *p* = 0.0246.

**FIGURE 5:**
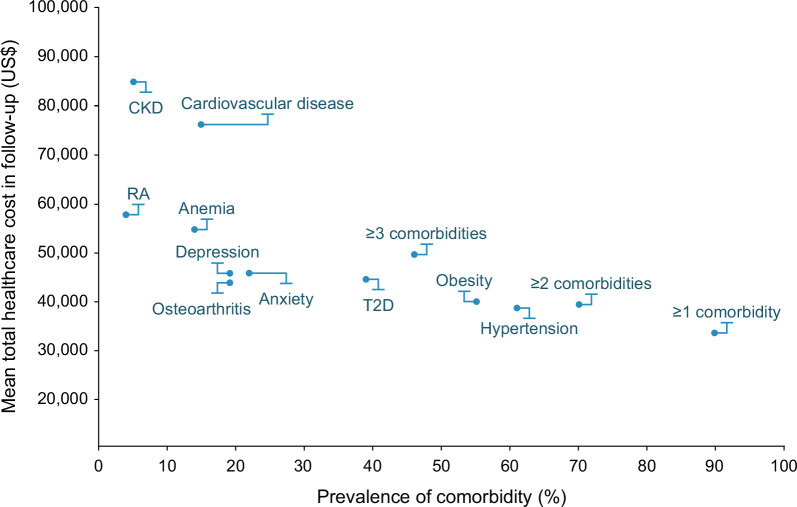
Total health care cost at follow-up by the prevalence of comorbidities in the no-cirrhosis group. When examining the health care costs for patients with multiple comorbidities, the following comorbidities were considered: anemia, anxiety, CKD, CVD, depression, hypertension, obesity, osteoarthritis, RA, and T2D. Abbreviation: T2D, type 2 diabetes.

The prevalence of comorbidities at baseline was significantly higher in patients with a high-cost burden compared with those with a non-high–cost burden (*p*<0.0001; Figure 4). This was also the case for CV-related comorbidities (all *p*<0.0001 except for cardiac arrest [*p* = 0.0246]; Figure 4). The use of glucose-lowering treatment (43% of patients with high-cost vs. 21% of patients with non-high–cost) and CV-related medication (72% vs. 58%, respectively) was higher in the high-cost group compared with the non-high–cost group. Table 2 presents the mean aDCSI and QCI scores and noninvasive test scores for patients in the high-cost group versus those in the non-high–cost group. The mean FIB-4 score was higher in the high-cost versus non-high–cost group (1.51 [207 patients] vs. 1.35 [351 patients], respectively; *p*<0.0001), along with the mean aspartate aminotransferase to platelet ratio (0.63 [272 patients] vs. 0.51 [453 patients], respectively; *p*<0.0001) and NAFLD fibrosis score (–1.15 [189 patients] vs. –1.60 [312 patients], respectively; *p* = 0.0001). Mean HbA1c (glycated hemoglobin) (7.01% and 6.55%) and BMI (35.18 and 34.54 kg/m^2^) were also numerically higher in the high-cost versus non-high–cost group, respectively. Patient characteristics associated with a high-cost burden in the cirrhosis group were also investigated and are included in the supplemental material (Supplemental Figure S1, http://links.lww.com/HC9/A977; Supplemental Table 2, http://links.lww.com/HC9/A977). The analysis of total health care follow-up costs in patients in the no-cirrhosis group, stratified by the prevalence of comorbidities (Figure 5), shows that patients with comorbidities such as chronic kidney disease and CVD had much higher follow-up costs than other comorbidities. The total follow-up costs increased as the number of comorbidities increased, with costs of $33,749, $39,556, and $49,789 for patients with 1, 2, and 3 comorbidities, respectively.

**TABLE 2:** Mean values for clinical biomarkers and measures of clinical burden in the no-cirrhosis group stratified into high-cost burden (≥$13,555) and non-high–cost burden (<$13,555)

	No cirrhosis		
	Non-high–cost (N=8957)	High-cost (N=5928)	*p*
MASH-relevant biomarkers			
APRI, n	516	313	0.2496
Mean (SD)	0.50 (0.50)	0.63 (0.93)	
Median (Q1, Q3)	0.37 (0.23, 0.59)	0.38 (0.23, 0.69)	
Fibrosis-4 index, n	392	246	
High (>2.67), n	26	28	
Intermediate (1.30–2.67), n	105	78	
Low (<1.30), n	261	140	
Mean (SD)	1.28 (1.01)	1.53 (1.38)	0.0228
Median (Q1, Q3)	1.03 (0.71, 1.46)	1.12 (0.72, 1.80)	
NAFLD fibrosis score, n	355	225	
Mean (SD)	–1.69 (1.56)	–1.09 (1.60)	0.0001
Median (Q1, Q3)	–1.70 (–2.75, –0.75)	–1.16 (–2.11, –0.1)	
Other clinical measures			
QCI			
Mean (SD)	1.44 (1.25)	2.17 (1.57)	<0.0001
Median (Q1, Q3)	2 (0, 2)	2 (1, 3)	
aDCSI			
Mean (SD)	0.46 (0.98)	1.10 (1.60)	<0.0001
Median (Q1, Q3)	0 (0, 0)	0 (0, 2)	
HbA1c, n	388	253	
Mean % (SD)	6.55 (1.60)	7.01 (1.78)	<0.0001
Median % (Q1, Q3)	6.10 (5.60, 7.00)	6.50 (5.80, 7.70)	
BMI, n	1871	1211	
Mean, kg/m^2^ (SD)	34.54 (6.46)	35.18 (6.64)	<0.0001
Median, kg/m^2^ (Q1, Q3)	32.93 (29.09, 37.86)	35.15 (30.54, 40.14)	

Abbreviations: aDCSI, adapted Diabetes Complications Severity Index; APRI, aspartate aminotransferase to platelet ratio; HbA1C, glycated hemoglobin; MASH, metabolic dysfunction–associated steatohepatitis; QCI, Quan-Charlson Index.

### Change in health care costs following MASH diagnosis

The total mean patient costs for all-cause disease at baseline and follow-up after MASH diagnosis, respectively, were $17,951 and $39,568 for the no-cirrhosis group and $43,118 and $98,574 for the cirrhosis group (Figure 6A). The mean (SD) increases in total patient costs from baseline following MASH diagnosis were $21,618 (973,377) for the no-cirrhosis group and $55,456 (430,133) for the cirrhosis group. Inpatient costs at follow-up accounted for the highest expenditure in both the no-cirrhosis and cirrhosis groups. When costs were analyzed according to liver-related causes, inpatient costs at follow-up accounted for the highest expenditure in the cirrhosis group, whereas outpatient costs were highest at follow-up in the no-cirrhosis group (Figure 6B). For CV-related causes, inpatient costs at follow-up accounted for the highest expenditure in both groups (Figure 6C). For liver- and CV-related causes, inpatient costs were substantially higher at follow-up versus baseline in the cirrhosis group (Figure 6D). For causes other than liver-and CV-related, outpatient costs at baseline and follow-up were the highest expenditure in both groups (Figure 6E). A breakdown of mean outpatient costs by health care expenditure category for the no-cirrhosis and cirrhosis group is provided in Supplemental Table S3,http://links.lww.com/HC9/A977.

FIGURE 6Mean costs in health care expenditure by all-cause disease (A), liver-related causes (B), cardiovascular-related causes (C), liver- and CV-related causes (D), and causes other than liver-and cardiovascular-related (E) for the no-cirrhosis and cirrhosis groups.

**Figure:**
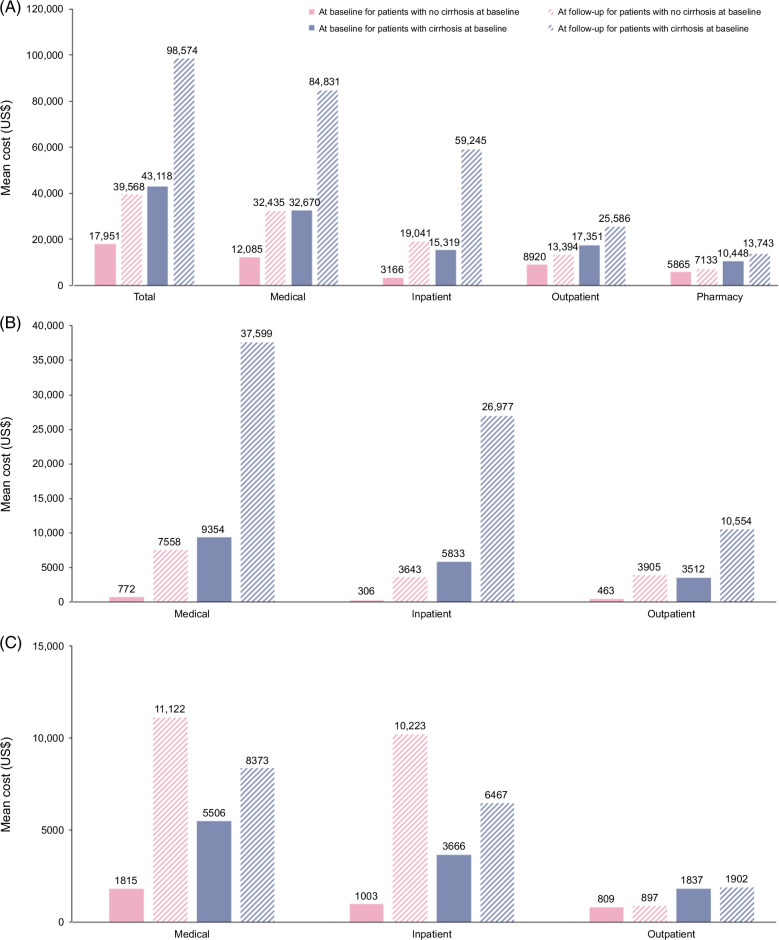

**Figure:**
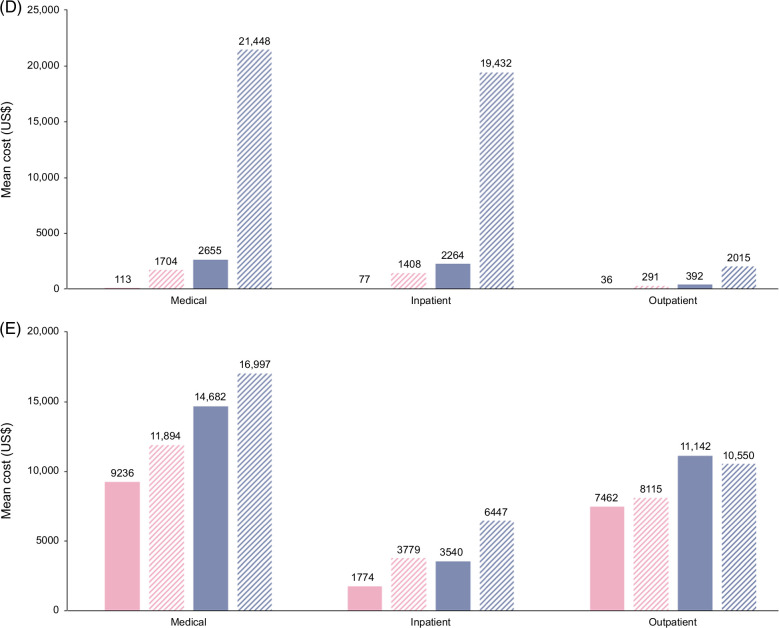

### Characteristics of patients in the no-cirrhosis group associated with higher change in total health care costs

In the no-cirrhosis group, greater increases in costs following MASH diagnosis were associated with the presence (vs. absence) of certain comorbidities (Supplemental Figure S2, http://links.lww.com/HC9/A977; Supplemental Table S4, http://links.lww.com/HC9/A977), including CVD (54% increase), rheumatoid arthritis (17%), anemia (25%), obesity (30%), and type 2 diabetes (14%). An increase in QCI also resulted in an increase in cost, with each unit increase resulting in a 16% cost increase. For each CV-related medication class prescribed, the cost increased by 4%.

## DISCUSSION

The economic burden in patients with MASH has not been extensively investigated to date, especially for those without cirrhosis. This retrospective, observational study aimed to understand demographic and clinical characteristics, economic burden, and factors influencing the economic burden in MASH.

The analysis demonstrated that most patients had a comorbidity at baseline, with a higher prevalence observed in those with cirrhosis compared with those without. In addition, the cirrhosis group had significantly higher annual total health care costs in the follow-up period compared with the no-cirrhosis group. This validates the findings from studies in populations with MASH^12^ and MASLD.^29^ The usage of glucose-lowering and CV-related medications in baseline was also greater in the cirrhosis versus no-cirrhosis group, which contributes to the higher economic burden associated with more severe disease. Preventing progression to cirrhosis and managing MASH-associated comorbidities may reduce the economic burden by mitigating costs associated with cirrhosis-related complications and MASH-related comorbidities.^30^

This study defined and characterized patients in the no-cirrhosis group with a high-cost burden, using the United States national estimate on annual health care expenditure as a threshold (≥$13,555). This threshold of ≥$13,555 was used to allow for a national-level benchmark (adjusted for 2023 values, the latest year of data availability). To the best of our knowledge, this topic and methodology have not been included in previous literature, and, as such, our study provides compelling new evidence on the economic burden of individuals at a less severe stage of disease. A significant proportion of the no-cirrhosis group had a cost burden exceeding $13,555, and this high-cost group had a higher prevalence and burden (as measured by QCI) of comorbidities than the non-high–cost group. Furthermore, certain comorbidities, such as CVD and chronic kidney disease, were associated with higher overall costs, regardless of the cost threshold, and greater increases in costs following MASH diagnosis, along with higher QCI and number of medications. This demonstrates that even in less advanced MASH, the presence of comorbidities is associated with higher costs and that there is a strong overlap in the characteristics that drive the economic and clinical burden in MASH.

The relationship between cardiometabolic diseases, such as MASH, type 2 diabetes, chronic kidney disease, and CVD, is not fully understood, but there is evidence of a bidirectional influence of these comorbidities on disease risk and severity,^20^,^21^,^22^ and CVD and MASH are strongly linked.^31^,^32^ In addition, Fishman et al^33^ recently reported that MASH comprises a high portion of total health care costs among patients with MASH and type 2 diabetes, with an incremental increase in costs per year of 42% (+$4692) when adding MASH to type 2 diabetes. As such, one may expect the worsening of some cardiometabolic comorbidities as MASH progresses, thereby further increasing the economic burden. In this study, CVD was associated with a 67% higher increase in costs following MASH diagnosis (vs. not having CVD). In a population of adults with type 2 diabetes and ischemic heart disease, worsening MASLD was associated with markers of CVD risk.^22^ Studies in patients with type 2 diabetes have also shown that patients with type 2 diabetes and MASH have a higher rate of liver-related adverse outcomes compared with those with type 2 diabetes and MASLD, indicating that the presence of type 2 diabetes worsens long-term outcomes in patients with MASLD.^20^ The presence of MASLD alongside type 2 diabetes has also been linked to increased probability of diabetic nephropathy, and more severe MASLD was associated with elevated markers of chronic kidney disease;^34^ for patients with chronic kidney disease, more severe liver steatosis was correlated with higher serum creatinine concentrations and lower estimated glomerular filtration rate.^35^ While it is challenging to understand the specific contribution of MASH to the overall cost burden, there is strong evidence from the current and other studies that MASH alone represents an increasing burden on health care that is exacerbated by comorbid conditions.^12^,^16^,^30^ Therefore, although the less advanced disease seen in the no-cirrhosis group could be expected to have consistently lower costs, the impact of comorbidities on the overall expense and the impact of MASH on such comorbidities should be considered when discussing the cost burden for the no-cirrhosis group and in clinical decision-making regarding disease management.

When looking at the driving factors for the increase in cost, this study found that inpatient costs were the biggest contributor, suggesting that hospitalization is a key driver of the increase in health care expenditure following MASH diagnosis. Pharmacy costs contributed little to the increase in cost, likely due to the lack of approved pharmacological therapies for MASH at the time of the study.^5^

MASH is often underdiagnosed or diagnosed at later stages.^7^,^8^ This is supported by the current finding that 12% of patients in this study had cirrhosis at the time of MASH diagnosis, indicating a delay in diagnosis. It is feasible that delayed diagnoses may have resulted in a high-cost burden due to an advanced disease stage, which could have been reduced if MASH had been diagnosed earlier. As seen in this analysis, the annualized mean cost following MASH diagnosis was twice as high for the cirrhosis versus no-cirrhosis group. Similar results have been reported in several studies that investigated MASLD and MASH severity and economic burden.^9^,^12^,^29^ An increase in health care costs was reported in a 2018 study^12^ of 3754 patients with MASH in which patients with advanced MASH (fibrosis stage 3–4) had higher costs versus early MASH (fibrosis stage 0–2). Indirect costs were also higher in advanced compared with early MASH.^12^ A 2010 study analyzed data from 976 patients with MASLD and found that costs were higher for patients with versus without cirrhosis.^29^ Costs were also higher for patients with decompensated versus compensated cirrhosis.^29^ Another study, which retrospectively analyzed data from 2007 to 2017, showed that patients with MASLD who had advanced fibrosis had higher costs.^9^ Noninvasive and minimally invasive tests for MASH components are being investigated as a way of providing information on disease severity,^36^,^37^ and could be used to analyze how cost burden changes as disease progresses or regresses. While the small sample size of patients with noninvasive test scores in this study means that the data should be interpreted with caution, there was a clear trend between a high-cost burden and worsening scores such as FIB-4, which builds on evidence of increased FIB-4 scores correlating with a higher cost burden.^9^

Proactive intervention(s) to delay, halt, or revert the progression of MASH and associated comorbidities could reduce the clinical and economic burden associated with MASH. These include changes in lifestyle and diet and administration of pharmacotherapy; however, at the time of the study, no MASH-specific medication was available, and an unmet need exists for new therapies. Moreover, broader collaborative action at national and international levels may be warranted to drive substantive economic benefits. In line with other chronic conditions, including obesity and type 2 diabetes,^38^,^39^ Allen et al^19^ have called for a global MASLD and MASH investment framework to guide the development of country-specific targets, optimize resource allocation, and reduce disease prevalence.^17^,^40^

A global consensus statement published in 2023 agreed to replace the terms NAFLD and NASH with MASLD and MASH, respectively, to reflect the increased understanding of the metabolic etiology of MASLD as well as to remove potentially stigmatizing language.^1^ This change in nomenclature was also accompanied by a change in definitions, with MASLD defined as the presence of at least 1 cardiometabolic risk factor relating to weight, glycemic dysregulation, blood pressure, or lipid dysregulation, in addition to steatosis.^1^ In the current study, we used the new nomenclature, although patients were identified and classified with the existing ICD codes for NASH. It is noteworthy that a recent global expert consensus paper on ICD coding in steatotic liver disease found a high degree of consensus that NAFLD and NASH ICD codes can be updated to reflect the new MASLD and MASH terminology, respectively, without a need for new codes.^41^

### Strengths

This study included a large sample size, capturing data and trends from real-world sources. There is a scarcity of data on health care costs associated with MASH, particularly for those without cirrhosis, and this study contributes to the analyses in this field. We also assessed the impact of various comorbidities on economic burden and provided a potential definition of high health care costs, which could be used to explore characteristics that are associated with this group. These findings complement the previous data on clinical burden in patients with MASH and could help guide treatment plans for individual patients, as well as provide broader recommendations to help reduce the clinical and economic burden of MASH.

### Limitations

As an observational study, limitations, such as missing data, mean that it is possible that not all patients with MASH were identified, and there was potential for misclassification of MASLD as MASH. Moreover, cirrhosis identification may have been inconsistent, with the likelihood that some patients in the no-cirrhosis group had cirrhosis, particularly due to its asymptomatic nature. However, the ICD codes used in the current study have been validated for the identification of cirrhosis in chronic liver disease cohorts along with diverse patient populations from Europe and North America, with high positive predictive values and sensitivity for the identification of cirrhosis.^42^,^43^ Furthermore, diagnosis of MASH through biopsy or noninvasive tests was not available, and it was not possible to identify the CV events that happened outside the facilities that were covered by the database used for the current study. However, the utilization of real-world data means that this study reflects patients diagnosed with MASH as seen in a real-world setting and still allows for the identification of important trends in the clinical and economic outcomes assessed in this study. Studies have shown that costs can be higher in the first year after diagnosis compared with subsequent years,^18^ so further studies are required to determine long-term costs and fluctuations over time. Currently, there is a need for a standard assessment of economic burden across MASLD and MASH, in terms of types of databases and modeling techniques, so it is challenging to draw comparisons across studies.^17^ Using the national mean health care expenditure, as was done for this study, could be used as a way of standardizing high- and non-high–cost thresholds across studies investigating the economic burden of MASH, with the caveat that differences in source data for US expenditure cost estimates and the source data for cost estimates in different models mean the data are not directly comparable.

This study contributes to economic health care burden data in the MASH population and the detailed understanding of the burden among those without cirrhosis and provides a definition of patients with a high-cost burden. Those with cirrhosis had a higher burden of comorbidity and CV-related and glucose-lowering medication use than those without. A diagnosis of MASH was associated with an increase in cost, largely driven by inpatient costs; the greatest increase was seen in the cirrhosis group. Nevertheless, a notable proportion of those without cirrhosis had a high-cost burden, among whom the burden of comorbidities was elevated compared with those below the high-cost burden threshold.

These results complement previous findings demonstrating that early diagnosis and prevention of disease progression could reduce the comorbidity and economic burden associated with MASH. In addition, the results illustrate the economic burden in noncirrhotic MASH and, together with previously published literature, indicate that factors contributing to increased economic burden are also clinically relevant, which may help to better manage this disease stage and reduce the burden on patients and health care systems.

## Supplementary Material

SUPPLEMENTARY MATERIAL
