# Supplementary material for: Estimating the economic impact of comorbidities in patients with MASH and defining high-cost burden in patients with noncirrhotic MASH
Source: Hepatol Commun. 2024 Jul 22;8(8):e0488. doi: 10.1097/HC9.0000000000000488 (PMC11265778; doi:10.1097/HC9.0000000000000488)
Supplement: SUPPLEMENTARY MATERIAL [file hc9-8-e0488-s001.docx]

**Supplemental Digital Content**

**Estimating the economic impact of comorbidities in patients with MASH and defining high-cost burden in patients with non-cirrhotic MASH: a retrospective, observational, cohort study (AWARE)**

Zobair M. Younossi, MD,^1,2*^ Kamal Kant Mangla, BTech,^3*^ Abhishek Shankar Chandramouli, BTech,^4*^ Jeffrey V. Lazarus, PhD^2,5,6^

^1^Beatty Liver and Obesity Research Program, Inova Health System, Falls Church, VA, USA

^2^The Global NASH Council, Washington DC, United States

^3^Novo Nordisk A/S, Søborg, Denmark

^4^Novo Nordisk Service Center India Pvt Ltd, Bangalore, India

^5^CUNY Graduate School of Public Health and Health Policy (CUNY SPH), New York, NY, USA

^6^Barcelona Institute for Global Health (ISGlobal), Hospital Clínic, University of Barcelona, Barcelona, Spain

*Affiliations during the study

**Correspondence:**

Zobair M. Younossi, Beatty Liver and Obesity Research Program, Claude Moore Health Education and Research Building, 3300 Gallows Road, Falls Church, VA 22042, USA; Tel: +1 703 776 2540; Email: zobair.younossi@cldq.org

Jeffrey V. Lazarus, CUNY Graduate School of Public Health and Health Policy, 55 West 125^th^ Street, New York, NY 10027, USA; Tel: +1 (646) 364-9600; Email: Jeffrey.Lazarus@SPH.CUNY.edu

**SDC TABLE 1** List of ICD codes for comorbidities

| **Diagnosis** | **ICD classification** | **ICD codes** |
| --- | --- | --- |
| Atrial fibrillation | ICD-10, ICD-10-CM, ICD-9-CM | I48, I48.0, I48.1, I48.11, I48.19, I48.2, I48.20, I48.21, I48.3, I48.4, I48.9, I48.91, I48.92, 427.3, 427.31, 427.32 |
| Angina pectoris | ICD-10, ICD-10-CM, ICD-9CM | I20, I20.0, I20.1, I20.8, I20.9, I23.7, I25.11, I25.110, I25.111, I25.118, I25.119, I25.7, I25.70, I25.700, I25.701, I25.708, I25.709, I25.71, I25.710, I25.711, I25.718, I25.719, I25.72, I25.720, I25.721, I25.728, I25.729, I25.73, I25.730, I25.731, I25.738, I25.739, I25.75, I25.750, I25.751, I25.758, I25.759, I25.76, I25.760, I25.761, I25.768, I25.769, I25.79, I25.790, I25.791, I25.798, I25.799, 413, 413.1, 413.9 |
| Atherosclerotic cardiovascular disease | ICD-10, ICD-10-CM, ICD-9-CM | G45, G45.0, G45.1, G45.2, G45.3, G45.4, G45.8, G45.9, I20, I20.0, I20.1, I20.8, I20.9, I21, I21.0, I21.01, I21.02, I21.09, I21.1, I21.11, I21.19, I21.2, I21.21, I21.29, I21.3, I21.4, I21.9, I21.A, I21.A1, I21.A9, I22, I22.0, I22.1, I22.2, I22.8, I22.9, I23, I23.0, I23.1, I23.2, I23.3, I23.4, I23.5, I23.6, I23.7, I23.8, I24, I24.0, I24.1, I24.8, I24.9, I25, I25.0, I25.1, I25.10, I25.11, I25.110, I25.111, I25.118, I25.119, I25.2, I25.3, I25.4, I25.41, I25.42, I25.5, I25.6, I25.7, I25.70, I25.700, I25.701, I25.708, I25.709, I25.71, I25.710, I25.711, I25.718, I25.719, I25.72, I25.720, I25.721, I25.728, I25.729, I25.73, I25.730, I25.731, I25.738, I25.739, I25.75, I25.750, I25.751, I25.758, I25.759, I25.76, I25.760, I25.761, I25.768, I25.769, I25.79, I25.790, I25.791, I25.798, I25.799, I25.8, I25.81, I25.810, I25.811, I25.812, I25.82, I25.83, I25.84, I25.89, I25.9, I60, I60.0, I60.00, I60.01, I60.02, I60.1, I60.10, I60.11, I60.12, I60.2, I60.20, I60.21, I60.22, I60.3, I60.30, I60.31, I60.32, I60.4, I60.5, I60.50, I60.51, I60.52, I60.6, I60.7, I60.8, I60.9, I61, I61.0, I61.1, I61.2, I61.3, I61.4, I61.5, I61.6, I61.8, I61.9, I62, I62.0, I62.00, I62.01, I62.02, I62.03, I62.1, I62.9, I63, I63.0, I63.00, I63.01, I63.011, I63.012, I63.013, I63.019, I63.02, I63.03, I63.031, I63.032, I63.033, I63.039, I63.09, I63.1, I63.10, I63.11, I63.111, I63.112, I63.113, I63.119, I63.12, I63.13, I63.131, I63.132, I63.133, I63.139, I63.19, I63.2, I63.20, I63.21, I63.211, I63.212, I63.213, I63.219, I63.22, I63.23, I63.231, I63.232, I63.233, I63.239, I63.29, I63.3, I63.30, I63.31, I63.311, I63.312, I63.313, I63.319, I63.32, I63.321, I63.322, I63.323, I63.329, I63.33, I63.331, I63.332, I63.333, I63.339, I63.34, I63.341, I63.342, I63.343, I63.349, I63.39, I63.4, I63.40, I63.41, I63.411, I63.412, I63.413, I63.419, I63.42, I63.421, I63.422, I63.423, I63.429, I63.43, I63.431, I63.432, I63.433, I63.439, I63.44, I63.441, I63.442, I63.443, I63.449, I63.49, I63.5, I63.50, I63.51, I63.511, I63.512, I63.513, I63.519, I63.52, I63.521, I63.522, I63.523, I63.529, I63.53, I63.531, I63.532, I63.533, I63.539, I63.54, I63.541, I63.542, I63.543, I63.549, I63.59, I63.6, I63.8, I63.81, I63.89, I63.9, I64, I65, I65.0, I65.01, I65.02, I65.03, I65.09, I65.1, I65.2, I65.21, I65.22, I65.23, I65.29, I65.3, I65.8, I65.9, I66, I66.0, I66.01, I66.02, I66.03, I66.09, I66.1, I66.11, I66.12, I66.13, I66.19, I66.2, I66.21, I66.22, I66.23, I66.29, I66.3, I66.4, I66.8, I66.9, I67, I67.0, I67.1, I67.2, I67.3, I67.4, I67.5, I67.6, I67.7, I67.8, I67.81, I67.82, I67.83, I67.84, I67.841, I67.848, I67.85, I67.850, I67.858, I67.89, I67.9, I68, I68.0, I68.1, I68.2, I68.8, I69, I69.0, I69.00, I69.01, I69.010, I69.011, I69.012, I69.013, I69.014, I69.015, I69.018, I69.019, I69.02, I69.020, I69.021, I69.022, I69.023, I69.028, I69.03, I69.031, I69.032, I69.033, I69.034, I69.039, I69.04, I69.041, I69.042, I69.043, I69.044, I69.049, I69.05, I69.051, I69.052, I69.053, I69.054, I69.059, I69.06, I69.061, I69.062, I69.063, I69.064, I69.065, I69.069, I69.09, I69.090, I69.091, I69.092, I69.093, I69.098, I69.1, I69.10, I69.11, I69.110, I69.111, I69.112, I69.113, I69.114, I69.115, I69.118, I69.119, I69.12, I69.120, I69.121, I69.122, I69.123, I69.128, I69.13, I69.131, I69.132, I69.133, I69.134, I69.139, I69.14, I69.141, I69.142, I69.143, I69.144, I69.149, I69.15, I69.151, I69.152, I69.153, I69.154, I69.159, I69.16, I69.161, I69.162, I69.163, I69.164, I69.165, I69.169, I69.19, I69.190, I69.191, I69.192, I69.193, I69.198, I69.2, I69.20, I69.21, I69.210, I69.211, I69.212, I69.213, I69.214, I69.215, I69.218, I69.219, I69.22, I69.220, I69.221, I69.222, I69.223, I69.228, I69.23, I69.231, I69.232, I69.233, I69.234, I69.239, I69.24, I69.241, I69.242, I69.243, I69.244, I69.249, I69.25, I69.251, I69.252, I69.253, I69.254, I69.259, I69.26, I69.261, I69.262, I69.263, I69.264, I69.265, I69.269, I69.29, I69.290, I69.291, I69.292, I69.293, I69.298, I69.3, I69.30, I69.31, I69.310, I69.311, I69.312, I69.313, I69.314, I69.315, I69.318, I69.319, I69.32, I69.320, I69.321, I69.322, I69.323, I69.328, I69.33, I69.331, I69.332, I69.333, I69.334, I69.339, I69.34, I69.341, I69.342, I69.343, I69.344, I69.349, I69.35, I69.351, I69.352, I69.353, I69.354, I69.359, I69.36, I69.361, I69.362, I69.363, I69.364, I69.365, I69.369, I69.39, I69.390, I69.391, I69.392, I69.393, I69.398, I69.4, I69.8, I69.80, I69.81, I69.810, I69.811, I69.812, I69.813, I69.814, I69.815, I69.818, I69.819, I69.82, I69.820, I69.821, I69.822, I69.823, I69.828, I69.83, I69.831, I69.832, I69.833, I69.834, I69.839, I69.84, I69.841, I69.842, I69.843, I69.844, I69.849, I69.85, I69.851, I69.852, I69.853, I69.854, I69.859, I69.86, I69.861, I69.862, I69.863, I69.864, I69.865, I69.869, I69.89, I69.890, I69.891, I69.892, I69.893, I69.898, I69.9, I69.90, I69.91, I69.910, I69.911, I69.912, I69.913, I69.914, I69.915, I69.918, I69.919, I69.92, I69.920, I69.921, I69.922, I69.923, I69.928, I69.93, I69.931, I69.932, I69.933, I69.934, I69.939, I69.94, I69.941, I69.942, I69.943, I69.944, I69.949, I69.95, I69.951, I69.952, I69.953, I69.954, I69.959, I69.96, I69.961, I69.962, I69.963, I69.964, I69.965, I69.969, I69.99, I69.990, I69.991, I69.992, I69.993, I69.998, I70, I70.0, I70.1, I70.2, I70.20, I70.201, I70.202, I70.203, I70.208, I70.209, I70.21, I70.211, I70.212, I70.213, I70.218, I70.219, I70.22, I70.221, I70.222, I70.223, I70.228, I70.229, I70.23, I70.231, I70.232, I70.233, I70.234, I70.235, I70.238, I70.239, I70.24, I70.241, I70.242, I70.243, I70.244, I70.245, I70.248, I70.249, I70.25, I70.26, I70.261, I70.262, I70.263, I70.268, I70.269, I70.29, I70.291, I70.292, I70.293, I70.298, I70.299, I70.3, I70.30, I70.301, I70.302, I70.303, I70.308, I70.309, I70.31, I70.311, I70.312, I70.313, I70.318, I70.319, I70.32, I70.321, I70.322, I70.323, I70.328, I70.329, I70.33, I70.331, I70.332, I70.333, I70.334, I70.335, I70.338, I70.339, I70.34, I70.341, I70.342, I70.343, I70.344, I70.345, I70.348, I70.349, I70.35, I70.36, I70.361, I70.362, I70.363, I70.368, I70.369, I70.39, I70.391, I70.392, I70.393, I70.398, I70.399, I70.4, I70.40, I70.401, I70.402, I70.403, I70.408, I70.409, I70.41, I70.411, I70.412, I70.413, I70.418, I70.419, I70.42, I70.421, I70.422, I70.423, I70.428, I70.429, I70.43, I70.431, I70.432, I70.433, I70.434, I70.435, I70.438, I70.439, I70.44, I70.441, I70.442, I70.443, I70.444, I70.445, I70.448, I70.449, I70.45, I70.46, I70.461, I70.462, I70.463, I70.468, I70.469, I70.49, I70.491, I70.492, I70.493, I70.498, I70.499, I70.5, I70.50, I70.501, I70.502, I70.503, I70.508, I70.509, I70.51, I70.511, I70.512, I70.513, I70.518, I70.519, I70.52, I70.521, I70.522, I70.523, I70.528, I70.529, I70.53, I70.531, I70.532, I70.533, I70.534, I70.535, I70.538, I70.539, I70.54, I70.541, I70.542, I70.543, I70.544, I70.545, I70.548, I70.549, I70.55, I70.56, I70.561, I70.562, I70.563, I70.568, I70.569, I70.59, I70.591, I70.592, I70.593, I70.598, I70.599, I70.6, I70.60, I70.601, I70.602, I70.603, I70.608, I70.609, I70.61, I70.611, I70.612, I70.613, I70.618, I70.619, I70.62, I70.621, I70.622, I70.623, I70.628, I70.629, I70.63, I70.631, I70.632, I70.633, I70.634, I70.635, I70.638, I70.639, I70.64, I70.641, I70.642, I70.643, I70.644, I70.645, I70.648, I70.649, I70.65, I70.66, I70.661, I70.662, I70.663, I70.668, I70.669, I70.69, I70.691, I70.692, I70.693, I70.698, I70.699, I70.7, I70.70, I70.701, I70.702, I70.703, I70.708, I70.709, I70.71, I70.711, I70.712, I70.713, I70.718, I70.719, I70.72, I70.721, I70.722, I70.723, I70.728, I70.729, I70.73, I70.731, I70.732, I70.733, I70.734, I70.735, I70.738, I70.739, I70.74, I70.741, I70.742, I70.743, I70.744, I70.745, I70.748, I70.749, I70.75, I70.76, I70.761, I70.762, I70.763, I70.768, I70.769, I70.79, I70.791, I70.792, I70.793, I70.798, I70.799, I70.8, I70.9, I70.90, I70.91, I70.92, I73.9, Z95.1, Z95.5, 410, 410.01, 410.02, 410.1, 410.11, 410.12, 410.2, 410.21, 410.22, 410.3, 410.31, 410.32, 410.4, 410.41, 410.42, 410.5, 410.51, 410.52, 410.6, 410.61, 410.62, 410.7, 410.71, 410.72, 410.8, 410.81, 410.82, 410.9, 410.91, 410.92, 411, 411.1, 411.8, 411.81, 411.89, 412, 413, 413.1, 413.9, 414, 414.01, 414.02, 414.03, 414.04, 414.05, 414.06, 414.07, 414.1, 414.11, 414.12, 414.19, 414.2, 414.3, 414.4, 414.8, 414.9, 430, 431, 432, 432.1, 432.9, 433, 433.01, 433.1, 433.11, 433.2, 433.21, 433.3, 433.31, 433.8, 433.81, 433.9, 433.91, 434, 434.01, 434.1, 434.11, 434.9, 434.91, 435, 435.1, 435.2, 435.3, 435.8, 435.9, 436, 437, 437.1, 437.2, 437.3, 437.4, 437.5, 437.6, 437.7, 437.8, 437.9, 438, 438.1, 438.11, 438.12, 438.13, 438.14, 438.19, 438.2, 438.21, 438.22, 438.3, 438.31, 438.32, 438.4, 438.41, 438.42, 438.5, 438.51, 438.52, 438.53, 438.6, 438.7, 438.8, 438.81, 438.82, 438.83, 438.84, 438.85, 438.89, 438.9, 440, 440.1, 440.2, 440.21, 440.22, 440.23, 440.24, 440.29, 440.3, 440.31, 440.32, 440.4, 440.8, 440.9, 443.8, 443.81, 443.82, 443.89, 443.9 |
| Atherosclerosis | ICD-10, ICD-10-CM, ICD-9-CM | I70, I70.0, I70.1, I70.2, I70.20, I70.201, I70.202, I70.203, I70.208, I70.209, I70.21, I70.211, I70.212, I70.213, I70.218, I70.219, I70.22, I70.221, I70.222, I70.223, I70.228, I70.229, I70.23, I70.231, I70.232, I70.233, I70.234, I70.235, I70.238, I70.239, I70.24, I70.241, I70.242, I70.243, I70.244, I70.245, I70.248, I70.249, I70.25, I70.26, I70.261, I70.262, I70.263, I70.268, I70.269, I70.29, I70.291, I70.292, I70.293, I70.298, I70.299, I70.3, I70.30, I70.301, I70.302, I70.303, I70.308, I70.309, I70.31, I70.311, I70.312, I70.313, I70.318, I70.319, I70.32, I70.321, I70.322, I70.323, I70.328, I70.329, I70.33, I70.331, I70.332, I70.333, I70.334, I70.335, I70.338, I70.339, I70.34, I70.341, I70.342, I70.343, I70.344, I70.345, I70.348, I70.349, I70.35, I70.36, I70.361, I70.362, I70.363, I70.368, I70.369, I70.39, I70.391, I70.392, I70.393, I70.398, I70.399, I70.4, I70.40, I70.401, I70.402, I70.403, I70.408, I70.409, I70.41, I70.411, I70.412, I70.413, I70.418, I70.419, I70.42, I70.421, I70.422, I70.423, I70.428, I70.429, I70.43, I70.431, I70.432, I70.433, I70.434, I70.435, I70.438, I70.439, I70.44, I70.441, I70.442, I70.443, I70.444, I70.445, I70.448, I70.449, I70.45, I70.46, I70.461, I70.462, I70.463, I70.468, I70.469, I70.49, I70.491, I70.492, I70.493, I70.498, I70.499, I70.5, I70.50, I70.501, I70.502, I70.503, I70.508, I70.509, I70.51, I70.511, I70.512, I70.513, I70.518, I70.519, I70.52, I70.521, I70.522, I70.523, I70.528, I70.529, I70.53, I70.531, I70.532, I70.533, I70.534, I70.535, I70.538, I70.539, I70.54, I70.541, I70.542, I70.543, I70.544, I70.545, I70.548, I70.549, I70.55, I70.56, I70.561, I70.562, I70.563, I70.568, I70.569, I70.59, I70.591, I70.592, I70.593, I70.598, I70.599, I70.6, I70.60, I70.601, I70.602, I70.603, I70.608, I70.609, I70.61, I70.611, I70.612, I70.613, I70.618, I70.619, I70.62, I70.621, I70.622, I70.623, I70.628, I70.629, I70.63, I70.631, I70.632, I70.633, I70.634, I70.635, I70.638, I70.639, I70.64, I70.641, I70.642, I70.643, I70.644, I70.645, I70.648, I70.649, I70.65, I70.66, I70.661, I70.662, I70.663, I70.668, I70.669, I70.69, I70.691, I70.692, I70.693, I70.698, I70.699, I70.7, I70.70, I70.701, I70.702, I70.703, I70.708, I70.709, I70.71, I70.711, I70.712, I70.713, I70.718, I70.719, I70.72, I70.721, I70.722, I70.723, I70.728, I70.729, I70.73, I70.731, I70.732, I70.733, I70.734, I70.735, I70.738, I70.739, I70.74, I70.741, I70.742, I70.743, I70.744, I70.745, I70.748, I70.749, I70.75, I70.76, I70.761, I70.762, I70.763, I70.768, I70.769, I70.79, I70.791, I70.792, I70.793, I70.798, I70.799, I70.8, I70.9, I70.90, I70.91, I70.92, I73.9, 440, 440.1, 440.2, 440.21, 440.22, 440.23, 440.24, 440.29, 440.3, 440.31, 440.32, 440.4, 440.8, 440.9, 443.9 |
| Cardiac arrest | ICD-10, ICD-10-CM, ICD-9-CM | I46, I46.0, I46.1, I46.2, I46.8, I46.9, 427.5 |
| Cardiac arrest (history) | ICD-10, ICD-10-CM, ICD-9-CM | I46, I46.0, I46.1, I46.2, I46.8, I46.9, 427.5 |
| Cerebrovascular disease | ICD-10, ICD-10-CM, ICD-9-CM | G45, G45.0, G45.1, G45.2, G45.3, G45.4, G45.8, G45.9, I60, I60.0, I60.00, I60.01, I60.02, I60.1, I60.10, I60.11, I60.12, I60.2, I60.20, I60.21, I60.22, I60.3, I60.30, I60.31, I60.32, I60.4, I60.5, I60.50, I60.51, I60.52, I60.6, I60.7, I60.8, I60.9, I61, I61.0, I61.1, I61.2, I61.3, I61.4, I61.5, I61.6, I61.8, I61.9, I62, I62.0, I62.00, I62.01, I62.02, I62.03, I62.1, I62.9, I63, I63.0, I63.00, I63.01, I63.011, I63.012, I63.013, I63.019, I63.02, I63.03, I63.031, I63.032, I63.033, I63.039, I63.09, I63.1, I63.10, I63.11, I63.111, I63.112, I63.113, I63.119, I63.12, I63.13, I63.131, I63.132, I63.133, I63.139, I63.19, I63.2, I63.20, I63.21, I63.211, I63.212, I63.213, I63.219, I63.22, I63.23, I63.231, I63.232, I63.233, I63.239, I63.29, I63.3, I63.30, I63.31, I63.311, I63.312, I63.313, I63.319, I63.32, I63.321, I63.322, I63.323, I63.329, I63.33, I63.331, I63.332, I63.333, I63.339, I63.34, I63.341, I63.342, I63.343, I63.349, I63.39, I63.4, I63.40, I63.41, I63.411, I63.412, I63.413, I63.419, I63.42, I63.421, I63.422, I63.423, I63.429, I63.43, I63.431, I63.432, I63.433, I63.439, I63.44, I63.441, I63.442, I63.443, I63.449, I63.49, I63.5, I63.50, I63.51, I63.511, I63.512, I63.513, I63.519, I63.52, I63.521, I63.522, I63.523, I63.529, I63.53, I63.531, I63.532, I63.533, I63.539, I63.54, I63.541, I63.542, I63.543, I63.549, I63.59, I63.6, I63.8, I63.81, I63.89, I63.9, I64, I65, I65.0, I65.01, I65.02, I65.03, I65.09, I65.1, I65.2, I65.21, I65.22, I65.23, I65.29, I65.3, I65.8, I65.9, I66, I66.0, I66.01, I66.02, I66.03, I66.09, I66.1, I66.11, I66.12, I66.13, I66.19, I66.2, I66.21, I66.22, I66.23, I66.29, I66.3, I66.4, I66.8, I66.9, I67, I67.0, I67.1, I67.2, I67.3, I67.4, I67.5, I67.6, I67.7, I67.8, I67.81, I67.82, I67.83, I67.84, I67.841, I67.848, I67.85, I67.850, I67.858, I67.89, I67.9, I68, I68.0, I68.1, I68.2, I68.8, I69, I69.0, I69.00, I69.01, I69.010, I69.011, I69.012, I69.013, I69.014, I69.015, I69.018, I69.019, I69.02, I69.020, I69.021, I69.022, I69.023, I69.028, I69.03, I69.031, I69.032, I69.033, I69.034, I69.039, I69.04, I69.041, I69.042, I69.043, I69.044, I69.049, I69.05, I69.051, I69.052, I69.053, I69.054, I69.059, I69.06, I69.061, I69.062, I69.063, I69.064, I69.065, I69.069, I69.09, I69.090, I69.091, I69.092, I69.093, I69.098, I69.1, I69.10, I69.11, I69.110, I69.111, I69.112, I69.113, I69.114, I69.115, I69.118, I69.119, I69.12, I69.120, I69.121, I69.122, I69.123, I69.128, I69.13, I69.131, I69.132, I69.133, I69.134, I69.139, I69.14, I69.141, I69.142, I69.143, I69.144, I69.149, I69.15, I69.151, I69.152, I69.153, I69.154, I69.159, I69.16, I69.161, I69.162, I69.163, I69.164, I69.165, I69.169, I69.19, I69.190, I69.191, I69.192, I69.193, I69.198, I69.2, I69.20, I69.21, I69.210, I69.211, I69.212, I69.213, I69.214, I69.215, I69.218, I69.219, I69.22, I69.220, I69.221, I69.222, I69.223, I69.228, I69.23, I69.231, I69.232, I69.233, I69.234, I69.239, I69.24, I69.241, I69.242, I69.243, I69.244, I69.249, I69.25, I69.251, I69.252, I69.253, I69.254, I69.259, I69.26, I69.261, I69.262, I69.263, I69.264, I69.265, I69.269, I69.29, I69.290, I69.291, I69.292, I69.293, I69.298, I69.3, I69.30, I69.31, I69.310, I69.311, I69.312, I69.313, I69.314, I69.315, I69.318, I69.319, I69.32, I69.320, I69.321, I69.322, I69.323, I69.328, I69.33, I69.331, I69.332, I69.333, I69.334, I69.339, I69.34, I69.341, I69.342, I69.343, I69.344, I69.349, I69.35, I69.351, I69.352, I69.353, I69.354, I69.359, I69.36, I69.361, I69.362, I69.363, I69.364, I69.365, I69.369, I69.39, I69.390, I69.391, I69.392, I69.393, I69.398, I69.4, I69.8, 432, 432.1, 432.9, 433, 433.01, 433.1, 433.11, 433.2, 433.21, 433.3, 433.31, 433.8, 433.81, 433.9, 433.91, 434, 434.01, 434.1, 434.11, 434.9, 434.91, 435, 435.1, 435.2, 435.3, 435.8, 435.9, 436, 437, 437.1, 437.2, 437.3, 437.4, 437.5, 437.6, 437.7, 437.8, 437.9, 438, 438.1, 438.11, 438.12, 438.13, 438.14, 438.19, 438.2, 438.21, 438.22, 438.3, 438.31, 438.32, 438.4, 438.41, 438.42, 438.5, 438.51, 438.52, 438.53, 438.6, 438.7, 438.8, 438.81, 438.82, 438.83, 438.84, 438.85, 438.89, 438.9 |
| Deep vein thrombosis | ICD-10, ICD-10-CM, ICD-9-CM | I26, I26.0, I26.01, I26.02, I26.09, I26.9, I26.90, I26.92, I26.93, I26.94, I26.99, I80.1, I80.10, I80.11, I80.12, I80.13, I80.2, I80.20, I80.201, I80.202, I80.203, I80.209, I80.21, I80.211, I80.212, I80.213, I80.219, I80.22, I80.221, I80.222, I80.223, I80.229, I80.23, I80.231, I80.232, I80.233, I80.239, I80.24, I80.241, I80.242, I80.243, I80.249, I80.25, I80.251, I80.252, I80.253, I80.259, I80.29, I80.291, I80.292, I80.293, I80.299, I82.4, I82.40, I82.401, I82.402, I82.403, I82.409, I82.41, I82.411, I82.412, I82.413, I82.419, I82.42, I82.421, I82.422, I82.423, I82.429, I82.43, I82.431, I82.432, I82.433, I82.439, I82.44, I82.441, I82.442, I82.443, I82.449, I82.45, I82.451, I82.452, I82.453, I82.459, I82.46, I82.461, I82.462, I82.463, I82.469, I82.49, I82.491, I82.492, I82.493, I82.499, I82.4Y, I82.4Y1, I82.4Y2, I82.4Y3, I82.4Y9, I82.4Z, I82.4Z1, I82.4Z2, I82.4Z3, I82.4Z9, 415.1, 415.11, 415.12, 415.13, 415.19, 451.1, 451.11, 451.19, 451.2, 453.4, 453.41, 453.42 |
| Deep vein thrombosis (history) | ICD-10, ICD-10-CM, ICD-9-CM | I26, I26.0, I26.01, I26.02, I26.09, I26.9, I26.90, I26.92, I26.93, I26.94, I26.99, I27.24, I27.82, I80.1, I80.10, I80.11, I80.12, I80.13, I80.2, I80.20, I80.201, I80.202, I80.203, I80.209, I80.21, I80.211, I80.212, I80.213, I80.219, I80.22, I80.221, I80.222, I80.223, I80.229, I80.23, I80.231, I80.232, I80.233, I80.239, I80.24, I80.241, I80.242, I80.243, I80.249, I80.25, I80.251, I80.252, I80.253, I80.259, I80.29, I80.291, I80.292, I80.293, I80.299, I82.4, I82.40, I82.401, I82.402, I82.403, I82.409, I82.41, I82.411, I82.412, I82.413, I82.419, I82.42, I82.421, I82.422, I82.423, I82.429, I82.43, I82.431, I82.432, I82.433, I82.439, I82.44, I82.441, I82.442, I82.443, I82.449, I82.45, I82.451, I82.452, I82.453, I82.459, I82.46, I82.461, I82.462, I82.463, I82.469, I82.49, I82.491, I82.492, I82.493, I82.499, I82.4Y, I82.4Y1, I82.4Y2, I82.4Y3, I82.4Y9, I82.4Z, I82.4Z1, I82.4Z2, I82.4Z3, I82.4Z9, I82.5, I82.50, I82.501, I82.502, I82.503, I82.509, I82.51, I82.511, I82.512, I82.513, I82.519, I82.52, I82.521, I82.522, I82.523, I82.529, I82.53, I82.531, I82.532, I82.533, I82.539, I82.54, I82.541, I82.542, I82.543, I82.549, I82.55, I82.551, I82.552, I82.553, I82.559, I82.56, I82.561, I82.562, I82.563, I82.569, I82.59, I82.591, I82.592, I82.593, I82.599, I82.5Y, I82.5Y1, I82.5Y2, I82.5Y3, I82.5Y9, I82.5Z, I82.5Z1, I82.5Z2, I82.5Z3, I82.5Z9, 415.1, 415.11, 415.12, 415.13, 415.19, 416.2, 451.1, 451.11, 451.19, 451.2, 453.4, 453.41, 453.42, 453.5, 453.51, 453.52, 453.72 |
| Heart failure | ICD-10, ICD-10-CM, ICD-9-CM | I11.0, I13.0, I13.2, I50, I50.0, I50.1, I50.2, I50.20, I50.21, I50.22, I50.23, I50.3, I50.30, I50.31, I50.32, I50.33, I50.4, I50.40, I50.41, I50.42, I50.43, I50.8, I50.81, I50.810, I50.811, I50.812, I50.813, I50.814, I50.82, I50.83, I50.84, I50.89, I50.9, 402.01, 402.11, 402.91, 404.01, 404.03, 404.11, 404.13, 404.91, 404.93, 428, 428.1, 428.2, 428.21, 428.22, 428.23, 428.3, 428.31, 428.32, 428.33, 428.4, 428.41, 428.42, 428.43, 428.9 |
| Hypertensive disease | ICD-10, ICD-10-CM, ICD-9-CM | I10, I10 , I11, I11.0, I11.9, I12, I12.0, I12.9, I13, I13.0, I13.1, I13.10, I13.11, I13.2, I13.9, I15, I15.0, I15.1, I15.2, I15.8, I15.9, 401, 401.1, 401.9, 402, 402.01, 402.1, 402.11, 402.9, 402.91, 403, 403.01, 403.1, 403.11, 403.9, 403.91, 404, 404.01, 404.02, 404.03, 404.1, 404.11, 404.12, 404.13, 404.9, 404.91, 404.92, 404.93, 405, 405.01, 405.09, 405.1, 405.11, 405.19, 405.9, 405.91, 405.99 |
| Hypertensive disease (history) | ICD-10, ICD-10-CM, ICD-9-CM | I10, I11, I11.0, I11.9, I12, I12.0, I12.9, I13, I13.0, I13.1, I13.10, I13.11, I13.2, I13.9, I15, I15.0, I15.1, I15.2, I15.8, I15.9, 401, 401.1, 401.9, 402, 402.01, 402.1, 402.11, 402.9, 402.91, 403, 403.01, 403.1, 403.11, 403.9, 403.91, 404, 404.01, 404.02, 404.03, 404.1, 404.11, 404.12, 404.13, 404.9, 404.91, 404.92, 404.93, 405, 405.01, 405.09, 405.1, 405.11, 405.19, 405.9, 405.91, 405.99 |
| Ischemic heart disease | ICD-10, ICD-10-CM, ICD-9-CM | I20, I20.0, I20.1, I20.8, I20.9, I21, I21.0, I21.01, I21.02, I21.09, I21.1, I21.11, I21.19, I21.2, I21.21, I21.29, I21.3, I21.4, I21.9, I21.A, I21.A1, I21.A9, I22, I22.0, I22.1, I22.2, I22.8, I22.9, I23, I23.0, I23.1, I23.2, I23.3, I23.4, I23.5, I23.6, I23.7, I23.8, I24, I24.0, I24.1, I24.8, I24.9, I25, I25.0, I25.1, I25.10, I25.11, I25.110, I25.111, I25.118, I25.119, I25.2, I25.3, I25.4, I25.41, I25.42, I25.5, I25.6, I25.7, I25.70, I25.700, I25.701, I25.708, I25.709, I25.71, I25.710, I25.711, I25.718, I25.719, I25.72, I25.720, I25.721, I25.728, I25.729, I25.73, I25.730, I25.731, I25.738, I25.739, I25.75, I25.750, I25.751, I25.758, I25.759, I25.76, I25.760, I25.761, I25.768, I25.769, I25.79, I25.790, I25.791, I25.798, I25.799, I25.8, I25.81, I25.810, I25.811, I25.812, I25.82, I25.83, I25.84, I25.89, I25.9, 410, 410.01, 410.02, 410.1, 410.11, 410.12, 410.2, 410.21, 410.22, 410.3, 410.31, 410.32, 410.4, 410.41, 410.42, 410.5, 410.51, 410.52, 410.6, 410.61, 410.62, 410.7, 410.71, 410.72, 410.8, 410.81, 410.82, 410.9, 410.91, 410.92, 411, 411.1, 411.8, 411.81, 411.89, 412, 413, 413.1, 413.9, 414, 414.01, 414.02, 414.03, 414.04, 414.05, 414.06, 414.07, 414.1, 414.11, 414.12, 414.19, 414.2, 414.3, 414.4, 414.8, 414.9 |
| Ischemic heart disease (history) | ICD-10, ICD-10-CM, ICD-9-CM | I20, I20.0, I20.1, I20.8, I20.9, I21, I21.0, I21.01, I21.02, I21.09, I21.1, I21.11, I21.19, I21.2, I21.21, I21.29, I21.3, I21.4, I21.9, I21.A, I21.A1, I21.A9, I22, I22.0, I22.1, I22.2, I22.8, I22.9, I23, I23.0, I23.1, I23.2, I23.3, I23.4, I23.5, I23.6, I23.7, I23.8, I24, I24.0, I24.1, I24.8, I24.9, I25, I25.0, I25.1, I25.10, I25.11, I25.110, I25.111, I25.118, I25.119, I25.2, I25.3, I25.4, I25.41, I25.42, I25.5, I25.6, I25.7, I25.70, I25.700, I25.701, I25.708, I25.709, I25.71, I25.710, I25.711, I25.718, I25.719, I25.72, I25.720, I25.721, I25.728, I25.729, I25.73, I25.730, I25.731, I25.738, I25.739, I25.75, I25.750, I25.751, I25.758, I25.759, I25.76, I25.760, I25.761, I25.768, I25.769, I25.79, I25.790, I25.791, I25.798, I25.799, I25.8, I25.81, I25.810, I25.811, I25.812, I25.82, I25.83, I25.84, I25.89, I25.9, Z95.1, Z95.5, 410, 410.01, 410.02, 410.1, 410.11, 410.12, 410.2, 410.21, 410.22, 410.3, 410.31, 410.32, 410.4, 410.41, 410.42, 410.5, 410.51, 410.52, 410.6, 410.61, 410.62, 410.7, 410.71, 410.72, 410.8, 410.81, 410.82, 410.9, 410.91, 410.92, 411, 411.1, 411.8, 411.81, 411.89, 412, 413, 413.1, 413.9, 414, 414.01, 414.02, 414.03, 414.04, 414.05, 414.06, 414.07, 414.1, 414.11, 414.12, 414.19, 414.2, 414.3, 414.4, 414.8, 414.9, |
| Myocardial infarction | ICD-10, ICD-10-CM, ICD-9-CM | I21, I21.0, I21.01, I21.02, I21.09, I21.1, I21.11, I21.19, I21.2, I21.21, I21.29, I21.3, I21.4, I21.9, I21.A, I21.A1, I21.A9, I22, I22.0, I22.1, I22.2, I22.8, I22.9, 410, 410.01, 410.02, 410.1, 410.11, 410.12, 410.2, 410.21, 410.22, 410.3, 410.31, 410.32, 410.4, 410.41, 410.42, 410.5, 410.51, 410.52, 410.6, 410.61, 410.62, 410.7, 410.71, 410.72, 410.8, 410.81, 410.82, 410.9, 410.91, 410.92 |
| Peripheral artery disease | ICD-10, ICD-10-CM, ICD-9-CM | I70.2, I70.20, I70.201, I70.202, I70.203, I70.208, I70.209, I70.21, I70.211, I70.212, I70.213, I70.218, I70.219, I70.22, I70.221, I70.222, I70.223, I70.228, I70.229, I70.23, I70.231, I70.232, I70.233, I70.234, I70.235, I70.238, I70.239, I70.24, I70.241, I70.242, I70.243, I70.244, I70.245, I70.248, I70.249, I70.25, I70.26, I70.261, I70.262, I70.263, I70.268, I70.269, I70.29, I70.291, I70.292, I70.293, I70.298, I70.299, I73.9, 440.2, 440.21, 440.22, 440.23, 440.24, 440.29, 440.4, 443.9 |
| Stroke | ICD-10, ICD-10-CM, ICD-9-CM | I61, I61.0, I61.1, I61.2, I61.3, I61.4, I61.5, I61.6, I61.8, I61.9, I63, I63.0, I63.00, I63.01, I63.011, I63.012, I63.013, I63.019, I63.02, I63.03, I63.031, I63.032, I63.033, I63.039, I63.09, I63.1, I63.10, I63.11, I63.111, I63.112, I63.113, I63.119, I63.12, I63.13, I63.131, I63.132, I63.133, I63.139, I63.19, I63.2, I63.20, I63.21, I63.211, I63.212, I63.213, I63.219, I63.22, I63.23, I63.231, I63.232, I63.233, I63.239, I63.29, I63.3, I63.30, I63.31, I63.311, I63.312, I63.313, I63.319, I63.32, I63.321, I63.322, I63.323, I63.329, I63.33, I63.331, I63.332, I63.333, I63.339, I63.34, I63.341, I63.342, I63.343, I63.349, I63.39, I63.4, I63.40, I63.41, I63.411, I63.412, I63.413, I63.419, I63.42, I63.421, I63.422, I63.423, I63.429, I63.43, I63.431, I63.432, I63.433, I63.439, I63.44, I63.441, I63.442, I63.443, I63.449, I63.49, I63.5, I63.50, I63.51, I63.511, I63.512, I63.513, I63.519, I63.52, I63.521, I63.522, I63.523, I63.529, I63.53, I63.531, I63.532, I63.533, I63.539, I63.54, I63.541, I63.542, I63.543, I63.549, I63.59, I63.6, I63.8, I63.81, I63.89, I63.9, I64, 431, 433.01, 433.11, 433.21, 433.31, 433.81, 433.91, 434.01, 434.11, 434.91 |
| Stroke (history) | ICD-10, ICD-10-CM, ICD-9-CM | I61, I61.0, I61.1, I61.2, I61.3, I61.4, I61.5, I61.6, I61.8, I61.9, I63, I63.0, I63.00, I63.01, I63.011, I63.012, I63.013, I63.019, I63.02, I63.03, I63.031, I63.032, I63.033, I63.039, I63.09, I63.1, I63.10, I63.11, I63.111, I63.112, I63.113, I63.119, I63.12, I63.13, I63.131, I63.132, I63.133, I63.139, I63.19, I63.2, I63.20, I63.21, I63.211, I63.212, I63.213, I63.219, I63.22, I63.23, I63.231, I63.232, I63.233, I63.239, I63.29, I63.3, I63.30, I63.31, I63.311, I63.312, I63.313, I63.319, I63.32, I63.321, I63.322, I63.323, I63.329, I63.33, I63.331, I63.332, I63.333, I63.339, I63.34, I63.341, I63.342, I63.343, I63.349, I63.39, I63.4, I63.40, I63.41, I63.411, I63.412, I63.413, I63.419, I63.42, I63.421, I63.422, I63.423, I63.429, I63.43, I63.431, I63.432, I63.433, I63.439, I63.44, I63.441, I63.442, I63.443, I63.449, I63.49, I63.5, I63.50, I63.51, I63.511, I63.512, I63.513, I63.519, I63.52, I63.521, I63.522, I63.523, I63.529, I63.53, I63.531, I63.532, I63.533, I63.539, I63.54, I63.541, I63.542, I63.543, I63.549, I63.59, I63.6, I63.8, I63.81, I63.89, I63.9, I64, I69.1, I69.10, I69.11, I69.110, I69.111, I69.112, I69.113, I69.114, I69.115, I69.118, I69.119, I69.12, I69.120, I69.121, I69.122, I69.123, I69.128, I69.13, I69.131, I69.132, I69.133, I69.134, I69.139, I69.14, I69.141, I69.142, I69.143, I69.144, I69.149, I69.15, I69.151, I69.152, I69.153, I69.154, I69.159, I69.16, I69.161, I69.162, I69.163, I69.164, I69.165, I69.169, I69.19, I69.190, I69.191, I69.192, I69.193, I69.198, I69.3, I69.30, I69.31, I69.310, I69.311, I69.312, I69.313, I69.314, I69.315, I69.318, I69.319, I69.32, I69.320, I69.321, I69.322, I69.323, I69.328, I69.33, I69.331, I69.332, I69.333, I69.334, I69.339, I69.34, I69.341, I69.342, I69.343, I69.344, I69.349, I69.35, I69.351, I69.352, I69.353, I69.354, I69.359, I69.36, I69.361, I69.362, I69.363, I69.364, I69.365, I69.369, I69.39, I69.390, I69.391, I69.392, I69.393, I69.398, I69.4, 431, 433.01, 433.11, 433.21, 433.31, 433.81, 433.91, 434.01, 434.11, 434.91 |

*Abbreviations*: ICD, International Classification of Diseases; ICD-9-CM, International Classification of Diseases, 9^th^ Revision, Clinical Modification;
ICD-10-CM, International Classification of Diseases, 10^th^ Revision, Clinical Modification.

# SDC TABLE 2 Mean values for clinical biomarkers and measures of clinical burden in the cirrhosis group stratified into high-cost burden (≥$13,555) and non-high-cost burden (<$13,555)

|  |  | **Cirrhosis** | |
| --- | --- | --- | --- |
|  |  | **Non-high cost**  **(n = 610)** | **High cost**  **(n = 1424)** |
| **Cinical biomarkers** | | | |
| NFS | n | 28 | 66 |
|  | Mean (SD) | 0.20 (1.95) | 1.62 (1.57) |
| FIB-4 | n | 32 | 75 |
|  | Mean (SD) | 2.95 (2.51) | 3.87 (2.98) |
| APRI | n | 38 | 98 |
|  | Mean (SD) | 0.91 (0.86) | 1.16 (1.35) |
| **Clinical burden scores** | | | |
| aDCSI | n | 610 | 1424 |
|  | Mean (SD) | 1.25 (1.78) | 2.19 (2.21) |
| QCI | n | 610 | 1424 |
|  | Mean (SD) | 3.57 (1.78) | 4.52 (2.00) |

High cost was defined as ≥$13,555, according to the US national estimate on annual healthcare expenditure.

Abbreviations: aDSCI, adapted Diabetes Complications Severity Index; APRI, AST [aspartate amine transferase] to Platelet Ratio Index; FIB-4, Fibrosis-4 Index; NFS, NAFLD (non-alcoholic fatty liver disease) Fibrosis Score; QCI, Quan-Charlson Index; SD, standard deviation; US, United States.

# SDC TABLE 3 Mean costs in outpatient healthcare expenditure by primary cause

|  | **All-cause  disease** | | **Liver-related** | | **Cardiovascular related** | | **Liver- and cardiovascular related** | | **Non-liver and non- cardiovascular related** | |
| --- | --- | --- | --- | --- | --- | --- | --- | --- | --- | --- |
|  | Baseline | Follow up | Baseline | Follow up | Baseline | Follow up | Baseline | Follow up | Baseline | Follow up |
| **Emergency room visit** | | | | | | | | | | |
| No cirrhosis | 440 | 560 | 22 | 78 | 32 | 43 | 2 | 21 | 380 | 415 |
| Cirrhosis | 678 | 963 | 121 | 268 | 91 | 117 | 25 | 64 | 438 | 513 |
| **Physician visit** | | | | | | | | | | |
| No cirrhosis | 1,389 | 1,583 | 46 | 330 | 42 | 48 | 1 | 8 | 1,291 | 1,189 |
| Cirrhosis | 1,692 | 1,861 | 215 | 603 | 100 | 90 | 12 | 27 | 1,348 | 1,129 |
| **Lab, pathology, radiology** | | | | | | | | | | |
| No cirrhosis | 1,933 | 2,392 | 200 | 717 | 82 | 222 | 8 | 44 | 1,616 | 1,512 |
| Cirrhosis | 3,211 | 4,719 | 858 | 2,373 | 91 | 234 | 73 | 408 | 2,024 | 1,659 |
| **Other procedures** | | | | | | | | | | |
| No cirrhosis | 4,623 | 8,007 | 159 | 2,426 | 593 | 648 | 22 | 184 | 3,746 | 4,614 |
| Cirrhosis | 10,884 | 16,501 | 1,977 | 6,528 | 1,333 | 1,340 | 249 | 1,386 | 6,933 | 6,765 |
| **Other outpatient** | | | | | | | | | | |
| No cirrhosis | 535 | 852 | 36 | 354 | 60 | 66 | 4 | 32 | 429 | 386 |
| Cirrhosis | 887 | 1,543 | 341 | 781 | 91 | 121 | 32 | 130 | 399 | 484 |

Data shown are US$.

# SDC TABLE 4 Increase in total annualized healthcare cost post-MASH diagnosis in the non-cirrhosis group by comorbidities, cardiovascular medication and QCI score

|  |  | **Cost (US$)** | **Percentage increase** | **Probability of difference** |
| --- | --- | --- | --- | --- |
| Anemia | No | 12,569 | 25% | <0.0001 |
|  | Yes | 15,694 |  | - |
| Anxiety | No | 12,893 | 19% | <0.0001 |
|  | Yes | 15,315 |  | - |
| Cardiovascular disease | No | 11,273 | 54% | <0.0001 |
|  | Yes | 17,378 |  | - |
| Depression | No | 12,857 | 19% | <0.0001 |
|  | Yes | 15,356 |  | - |
| Diabetic neuropathy | No | 13,440 | 9% | 0.0579 |
|  | Yes | 14,707 |  | - |
| Hyperlipidemia | No | 15,030 | -13% | <0.0001 |
|  | Yes | 13,145 |  | - |
| Hypertensive disease | No | 12,742 | 22% | <0.0001 |
|  | Yes | 15,489 |  | - |
| Obesity | No | 12,324 | 30% | <0.0001 |
|  | Yes | 15,991 |  | - |
| Osteoarthritis | No | 13,111 | 15% | <0.0001 |
|  | Yes | 15,068 |  | - |
| Renal disease | No | 14,040 | 0% | 0.9513 |
|  | Yes | 14,084 |  | - |
| Gender | Male | 13,236 | 13% | <0.0001 |
|  | Female | 14,929 |  | - |
| Rheumatoid arthritis | No | 12,983 | 17% | 0.0073 |
|  | Yes | 15,212 |  | - |
| Type 2 diabetes | No | 13,148 | 14% | <0.0001 |
|  | Yes | 15,027 |  | - |
| Cardiovascular medication | - | 369* | 4% | - |
| QCI score | - | 3358* | 16% | - |

*Cost increase.
Abbreviations: QCI, Quan-Charlson Index

# SDC FIGURE 1 Prevalence of baseline comorbidities in the cirrhosis group with high-cost burden (≥$13,555) and non-high-cost burden (<$13,555)


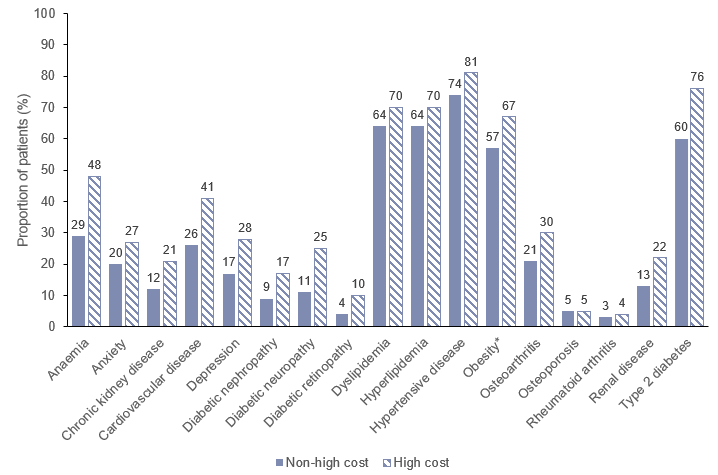


High cost was defined as ≥$13,555, according to the US national estimate on annual healthcare expenditure.

^a^Obesity classified as diagnosed or measured BMI ≥30 kg/m^2^.

Abbreviations: BMI, body mass index.

# SDC FIGURE 2 Increase in total annualized healthcare cost post-MASH diagnosis in the non-cirrhosis group by comorbidities, cardiovascular medication and QCI score


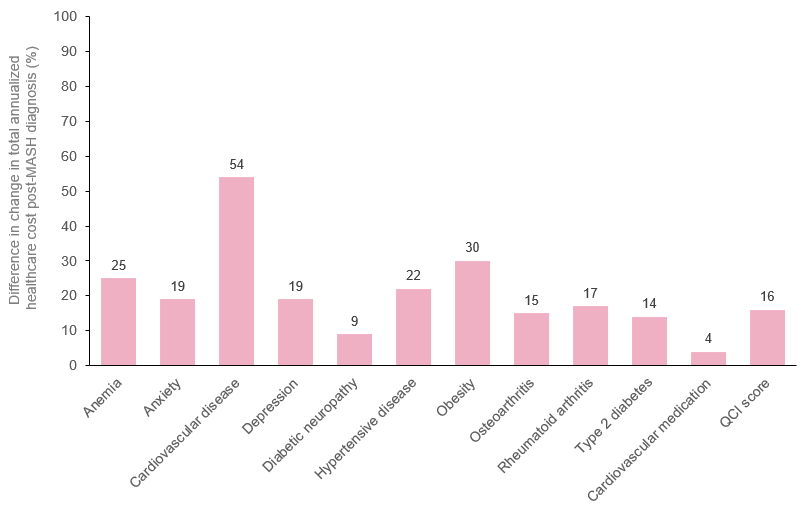


Difference is based on presence vs absence of comorbidity, or per unit increase for cardiovascular medication and QCI score.

Abbreviations: QCI, Quan-Charlson Index
